# A Bone in the Larynx for Eight Months—Spontaneous Expulsion and Recovery

**Published:** 1879-11

**Authors:** E. Fletcher Ingals

**Affiliations:** 188 Clark St., Chicago


### Clinical Reports.

### NOTES FROM PRIVATE PRACTICE.

### ARTICLE VI.

*A Bone in the Larynx for Eight Months—Spontaneous Expulsion and Recovery.*

Mr. S. E., æt 46, single, cook, applied last January at my clinic for diseases of the chest and throat, in the Central Free Dispensary, in consequence of hoarseness, dyspnœa and spasmodic cough.

An examination with the laryngoscope revealed a state of general congestion of the larynx with a diffused swelling about the size of a hazelnut, involving the right ventricular band, ary-epiglottic fold and right half of the posterior wall of the pharynx. The throat was so sensitive that a thorough examination was impossible.

Local astringent applications were made, and anodynes were given to relieve the severity of the cough, and chlorate of potassa was freely administered internally. After three or four weeks, the swelling continuing, and a hard external swelling also appearing over the right side of the larynx, it was thought best to place the patient on anti-syphylitic treatment, as his history pointed to a specific origin for the laryngeal trouble. The patient seemed to improve on this treatment, but after a few weeks the difficulty with his throat increased, and as he was unable to work and had no home, he was sent to the Alexian Brothers' Hospital, where he was under the care of Drs. Baxter and Hooper.

The external swelling was opened, but discharged little; the opening, however, did not heal. The patient left the hospital for a time, but soon returned again, and nearly all the time until

July, continued to take iodide of potassium, and occasionally tonics, until the 29th of the month, when, during a spasmodic cough, he expectorated a fragment of bone which seemed to be part of the clavicle of a barn-yard fowl. The bone was an inch and a quarter in length and quite sharp at one of its extremities.

The patient gave no history of having swallowed a bone, and he has not the slightest recollection of the accident. After the expulsion of the bone, the external wound in the neck promptly healed, the coughing ceased and convalescence was established so that the patient was discharged in a few days.

I saw the patient ten days after the bone had been expelled, and found him in perfect health with no evidence of his former disease excepting slight induration about the external cicatrix, and a diffused swelling of the right half or two-thirds of the posterior pharyngeal wall and slight swelling of the right side of the larynx.

This bone must have lodged in the pharyngo-laryngeal sinus of the right side, in which position foreign bodies of this character, as pins, fish-bones, etc., are very apt to be entangled. It is not singular that the bone remained so long, but it seems almost incredible that a person should get a foreign body of its character into the throat without any knowledge of the accident.

E. Fletcher Ingals, m.d.

188 Clark St., Chicago.

